# On the Determining Cause of Labor

**Published:** 1872-06

**Authors:** C. C. P. Clark

**Affiliations:** Oswego, N. Y.


*On the Determining Cause of Labor.* By C. C. P. Clark, M.D., Oswego, N. Y.

The question of the determining cause of labor has been variously answered. A summary of the different solutions may be found in Cazeaux, pp. 386, *et seq.* An additional theory has been lately discussed in this journal.* Objections so obvious and conclusive have been made to each of them, and will indeed suggest themselves to every intelligent and reflecting mind, that they may be safely laid aside as hardly worthy of consideration or argument. The theory of M. Brown-Sequard, that an excess of venous blood in the organ at term causes the uterus to contract, cannot, it is true, be disproved, but it is so far-fetched, its elements are, to common physiological apprehension, so foreign to the matter to which he applies them, and the application is so unexpected, that it bears the stamp of an ingenious conceit rather than of a true interpretation of nature's simplicity and directness. No sound theory in any science was ever proposed that experts did not exclaim: "How simple! I wonder that I did not think of that before,"—so nearly are discoveries in science always allied to what was known before.

This leaf in the volume of nature has been studied as though human physiology was the entire treatise, and not a single chapter, which, to be fully understood, must be taken in connection with the whole scope of the work. An illustration from another part of that volume will throw light on this question. The husk, the seed case of a nut, and the decidua of a mammal, are physiologically identical. The functions, and therefore the life of each, are

* American Journal of Obstetrics and Diseases of Women and Children, Vol. IV, Nos. 1 and 2, May and August, 1871.

limited to a brief period; and as the husk or burr of the nut, when ripe, spontaneously forsakes the parent stock, so does the decidua of the human female. The phenomena of this process may be studied and described in detail, but the physiological law is ultimate and beyond analysis.

The investment of the human ovum, the temporary root and support of its individual life, is limited in the duration of its vitality to about 280 days, as in the mare it is to 330 days, and in the chestnut to 115 days. The burr of the nut may fall a few days sooner or a few days later, and so may the decidua of the female mammal. The invested fœtus, like the invested nut, has another law of life. In one sense it has an existence independent of the mother. Were it practically possible to warm and nourish it at any other hearth, it might develop and come to maturity in a foreign place, as the egg of a hen does in an artificial oven. The short-lived decidua, the feeding ground of the fœtus, belongs to the mother. Like the pulp of fruit in the botanical kingdom it perishes when mature, though its contents are destined to a longer vitality. The husk of the nut, the pulp of the apple, and the decidua of the human female, are destined when ripe, when their allotted functions are performed, to perish and decay.

The knowledge of this great physiological fact is alone sufficient to suggest that it is to the maturity of the decidua that we must look for the cause of the occurrence of labor at a fixed time. But the question arises: What is the particular change in the relations between the decidua and the uterus that now awakens reflex action, and precipitates labor? What is it that comes so suddenly to disturb the harmony that has so long existed, and substitute for the placidity of mere being a storm of physiological action? How does that which for months has been pleasing to the womb, now become offensive? To-day, all is peace; to-morrow, seeming repugnance, violence, pain. What is it that precipitates the change? Why does it occur to-day and not yesterday? This is the question of the determining cause of labor.

In the investigation of nature we need not always be satisfied with observing her; we may sometimes question her. Those branches of science which concern the human family do not always permit free questioning of their subject. Humanity often stands in the way. In this case, fortunately, crucial questioning is not only possible, but is daily practiced.

When the obstetrician, in a case of contracted pelvis for instance, desires to precipitate labor, to what class of measures does he resort to accomplish his purpose? By the bougie or the uterine douche he separates the decidua from the uterine wall over a certain space. The effect of such separation can be nothing else than to damage or destroy the vitality of the detached part. Dependent as that membrane is for its nourishment on the subjacent uterine surface, the life of the separated part must soon cease; nor can it be doubted that in a part of such low vitality the morbid condition and subsequent death induced by the separation would rapidly spread beyond the boundaries of the original injury, and result in further detachment. A perforating wound of the membranes being incapable of healing, would soon become an ulcer, and by the extension to its environment of the morbid action of which it must itself speedily become the seat, would in like manner lead to their cleavage from the uterine wall over a gradually extending area. Hence the sure influence of such puncture in inducing labor. The same reasoning applies to the effect of the introduction of a tent within the os uteri, and to all other modes of damaging the decidua.

Thus may labor be artificially precipitated. There is abundant reason for believing that at term a *spontaneous separation of the decidua* occurs, and is the *determining cause* of labor. The investment of the ovum is then mature. This ripeness, like the ripeness of fruit, is accompanied by a suspension of nutrition, and involves the loosening and detachment of this temporary organism from its seat. This separation, by the same analogy, and by the reason of the case, may be expected to begin in the least highly organized part of the investment. The decidual membrane, and not the highly vascular part of the decidua which helps to make up the placenta, is the first to yield up its life and let go its hold.

It will be noticed as an actual fact, on introducing the finger through the os uteri at an early stage of labor, and passing it in different directions between the decidual and the uterine surfaces, that a complete separation of those surfaces, as far as the finger can reach, has already taken place. Delicate manipulation will easily distinguish between actual separation and separability. This detachment will often be found to have taken place before the yielding of the os and neck can have produced it mechanically. Sometimes it exists in one direction from the os and not in another, and sometimes it will not be found to any considerable extent in any direction.

In these last cases subsequent phenomena will indicate that the cleavage, which at the beginning of labor is probably partial, has begun in a region removed from the os uteri. The watery oozing from that opening which commonly precedes, and still more uniformly attends the beginning of labor, and which has its source in the bared internal surface of the womb, or in the debris of the dead decidua, will not take place till a later period of the labor; then, when the region of detachment by gradual extension comes to communicate with the os uteri, this ooze will be noticed to come for a time in successive small gushes with the pains. This devitalization and decay of the decidua at a distant part, while in the neighborhood of the os it is still comparatively sound, explains what Cazeaux declares to be "nearly inexplicable," viz., that sometimes the membranes are ruptured high up instead of at the uterine orifice.

The spontaneous escape of the liquor amnii near term, but before the occurrence of labor pains, can only be due to the disintegration by over-ripeness of the decidual structure, and must imply some extent of detachment. Therefore it is that such escape, whether complete or incomplete, is soon followed by labor. The extreme bulging of the bag of waters before the head which is often noticed, is permitted by a detachment of the membranes largely in excess of the degree of dilatation of the os, and is not explicable on any other than the theory under consideration. That this spontaneous cleavage, variable in extent and seat, is among the phenomena of labor, is also clearly indicated by the fact that while, in withdrawing the placenta from the vagina, its accompanying membranes will *generally* be felt to have no adherence to the maternal parts, a considerable amount of force will *sometimes* be required to peel them away. Occasionally a portion of them will be left still clinging to the uterine walls, and the whole might be expected so to remain but that ripeness and death loosen their hold. Before term the decidua adheres to the uterine walls with such tenacity that it is impossible to suppose that its detachment is due to the mechanics of labor.

The doctrine of the partial physiological cleavage of the decidua from the uterus before labor, being thus sustained by analogy, by reason and by facts, may be deemed irrefragable. It may to some readers appear so obviously true as to make this argument seem superfluous. I have given it at some length, because this phenomenon is not put down among the possible causes of labor by any authors that have fallen into my hands.

The partially devitalized and detached decidua induces labor pains, because it thus *becomes a foreign body in contact with the inner surface of the uterus*, and as such is offensive to the reflex instincts of that organ. Why any foreign body in that situation excites reflex action is incapable of explanation. It is an ultimate physiological law. That the muscular power of the uterus, dormant perhaps through all its previous history, should thus suddenly be called into action, is no more strange than that the fœtus, shortly after birth, should for the first time void the meconium. The womb, like the rectum, is but a recess in a conduit to the outer world.

It helps us to believe that partial separation of the decidua is the normal provocation of labor when we remember that the pregnant womb is wonderfully insensible to anything amiss within it other than the contact of a foreign body. Witness the immense dilatation that it will bear from multiple fœtation, or from hydrops: witness the violent struggles of its inmate that it patiently submits to. It will bear for a long time undisturbed the death of the ovum. We sometimes see a fœtus expelled in an advanced stage of decomposition. A twin fœtus, as recently happened under my observation, may lie there, as shown by its stage of development, for months, and slowly undergo such changes as profoundly to deprave the blood and affect the health of the mother, without awakening the self-preservative instincts of that organ. So we see the husk adhere to the branch though the enclosed nut be abortive and dead. The husk belongs to the tree, but the nut belongs to the husk. But when at length the vitality of the decidual membrane is so far impaired as to loosen its hold upon its nutrient seat, then the quick is touched by the dead, and the blind and sleeping law of self-preservation awakens to its duty. The distant fœtus—vitally and physiologically distant—may fare well or ill; that matters little; but the womb will take care of itself, and when that investment of its transient inmate with which its walls are in contact, and of whose presence alone its reflex instincts are conscious, begins by separation to become a foreign body, it must be expelled.

There is room for doubt whether any pathological change in the decidua will bring on labor until it has first resulted in the partial detachment of that organ. Extreme fatty degeneration of the placenta, extending doubtless throughout the decidua, and other morbid conditions, are known to be compatible with muscular quietude of the uterus. It is therefore probable that the changes in structure which the maturity of the decidua involves, have nothing to do directly with the occurrence of labor. They are merely a *causa causarum.* Labor normally comes on, then and then only, when the decidua, cleaving from the uterine walls through ripeness, becomes a *quasi* foreign body in the cavity of the womb.

P. S.—Since this article was written, my friend, Dr. C. Macfarlane, has directed my attention to a paragraph on page 311 of the first volume of the American edition of Simpson's Obstetrical Works, where I find that I have been anticipated, in the doctrine here advanced, by that distinguished obstetrician. As it is, however, only incidentally touched upon by him, and does not appear to have generally attracted the attention of the profession, this fuller exposition of it may be not profitless.—*American Journal of Obstetrics, etc.*

